# Supplementary material for: MXene–Protein Corona Interfaces for Molecular Profiling of Alzheimer’s Disease
Source: bioRxiv. 2026 May 18:2026.05.14.725150. Preprint. [Version 1] doi: 10.64898/2026.05.14.725150 (PMC13228322; doi:10.64898/2026.05.14.725150)
Supplement: Supplement 1 [file media-1.pdf]

MXene–Protein Corona Interfaces for Molecular Profiling of Alzheimer's Disease

Samantha Velazquez[1], Matthew C. Juber[2], Dalton Brindley[3], Anupma Thakur[4], Babak Anasoori[4,5*], Edward Lau[2*], Ali Akbar Ashkarran[3,6*]

[1]Department of Biology, University of Colorado Colorado Springs, Colorado Springs, CO 80918, USA

[2]Department of Medicine, University of Colorado School of Medicine, Aurora, CO 80045, USA

[3]Department of Physics and Energy Science, University of Colorado Colorado Springs, Colorado Springs, CO 80918, USA

[4]School of Materials Engineering, Purdue University, West Lafayette, IN 47907, USA

[5]School of Mechanical Engineering, Purdue University, West Lafayette, IN 47907, USA

[6]BioFrontiers Center, University of Colorado Colorado Springs, Colorado Springs, CO 80918, USA

***Corresponding authors:** (BA, EL, AAA) emails: (banasori@purdue.edu; edward.lau@cuanschutz.edu; aashkarr@uccs.edu)

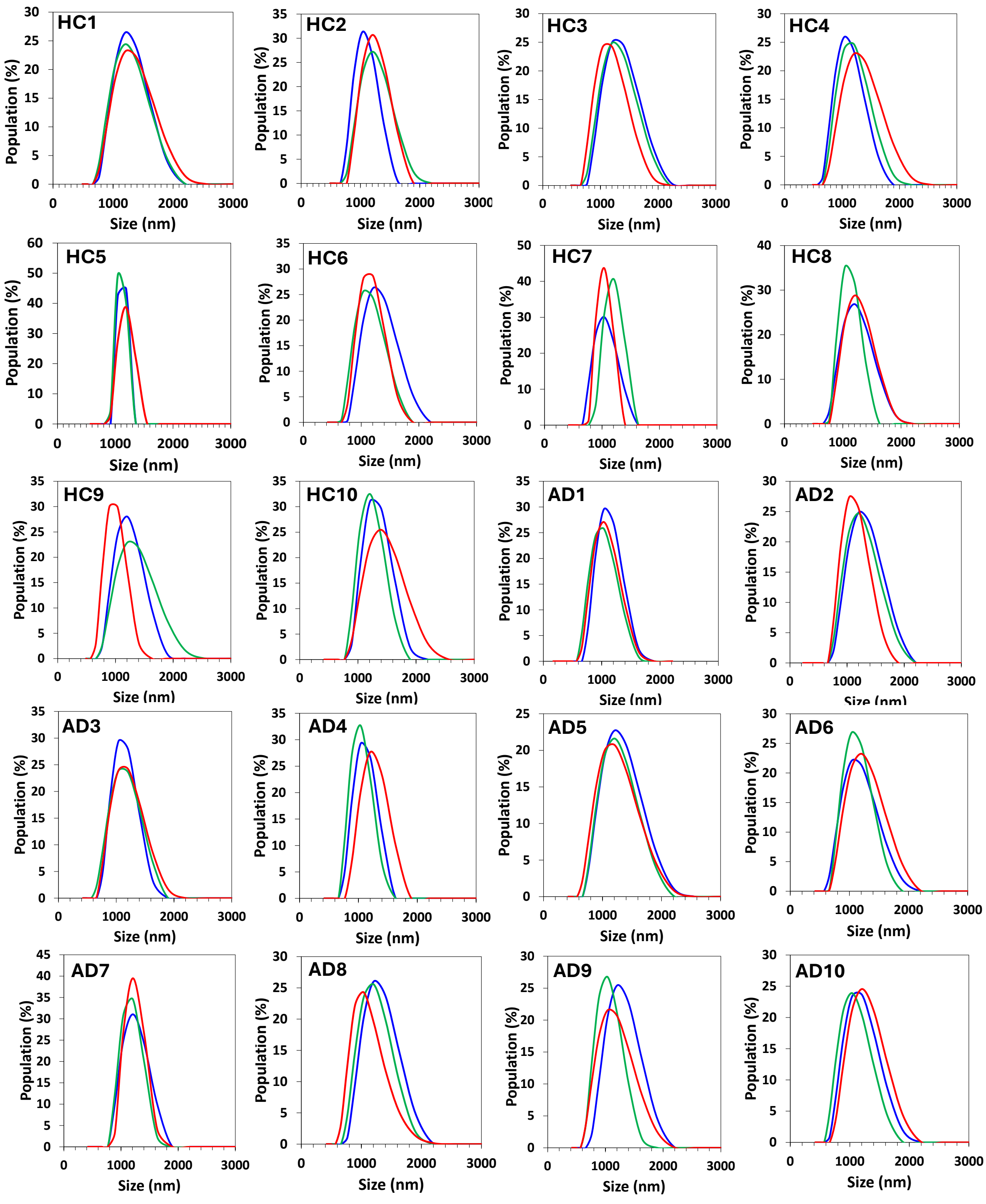

Figure S1: DLS analysis of $Ti_3C_2T_x$ MXene–PC complexes incubated with plasma from Alzheimer's disease (AD) patients and healthy controls (HC). Each curve represents the hydrodynamic size distribution of 2D $Ti_3C_2T_x$ MXene nanostructures following incubation with individual plasma samples ($n = 10$ per group). Different colors correspond to three independent repeats.

Table S1: Summary of average size, polydispersity index (PDI), zeta potential (n = 10 per group, three technical replicates per sample), and the corresponding standard deviation (SD) values of MXene–PC complexes formed with plasma from AD patients and HC.

MXene–PC Sample	DLS hydrodynamic Size (nm)	SD (nm)	PDI	DLS flake Size (nm)	SD (nm)	SD	Zeta potential (mV)	SD (mV)
**Bare**	1095.7	80.1	0.584	2538.8	278.4	0.07	-35.7	0.6
**HC-PC-1**	1272.7	24.7	0.289	3178.2	92.5	0.063	-24.2	0.3
**HC-PC-2**	1176.5	73.3	0.399	2824.8	264	0.014	-21.6	1.2
**HC-PC-3**	1253.8	69.1	0.378	3107.7	256.9	0.137	-27.6	0.1
**HC-PC-4**	1191.5	81.9	0.407	2879	296.8	0.075	-25.0	0.7
**HC-PC-5**	1173.1	45.5	0.323	2812.6	163.6	0.016	-24.0	0.4
**HC-PC-6**	1187.0	78.3	0.303	2862.7	283.3	0.037	-24.4	0.7
**HC-PC-7**	1202.3	117.6	0.366	2918.2	428.2	0.038	-24.6	0.1
**HC-PC-8**	1192.8	62.8	0.331	2883.7	227.7	0.058	-22.0	1.1
**HC-PC-9**	1164.3	139.3	0.399	2781	499.1	0.041	-24.0	0.1
**HC-PC-10**	1274.6	83.9	0.688	3185.4	314.5	0.011	-19.4	0.6
**AD-PC-1**	1152.5	38.5	0.407	2738.8	137.2	0.065	-23.7	0.2
**AD-PC-2**	1206.9	67.1	0.418	2935	244.8	0.042	-23.9	0.6
**AD-PC-3**	1125.4	21.1	0.372	2642.8	74.3	0.037	-21.6	0.1
**AD-PC-4**	1121.5	89.8	0.399	2629	315.8	0.015	-25.5	0.2
**AD-PC-5**	1235.4	31.2	0.314	3039.6	115.1	0.026	-20.6	0.1
**AD-PC-6**	1156.6	51.1	0.473	2753.4	182.5	0.050	-26.3	0.3
**AD-PC-7**	1204.3	27.4	0.380	2925.5	99.8	0.048	-23.4	0.5
**AD-PC-8**	1186.1	83.4	0.494	2859.4	301.6	0.102	-25.7	0.5
**AD-PC-9**	1143.2	93.4	0.691	2705.7	331.6	0.037	-22.1	0.1
**AD-PC-10**	1152.7	74.4	0.430	2739.5	265.2	0.116	-23.00	0.5

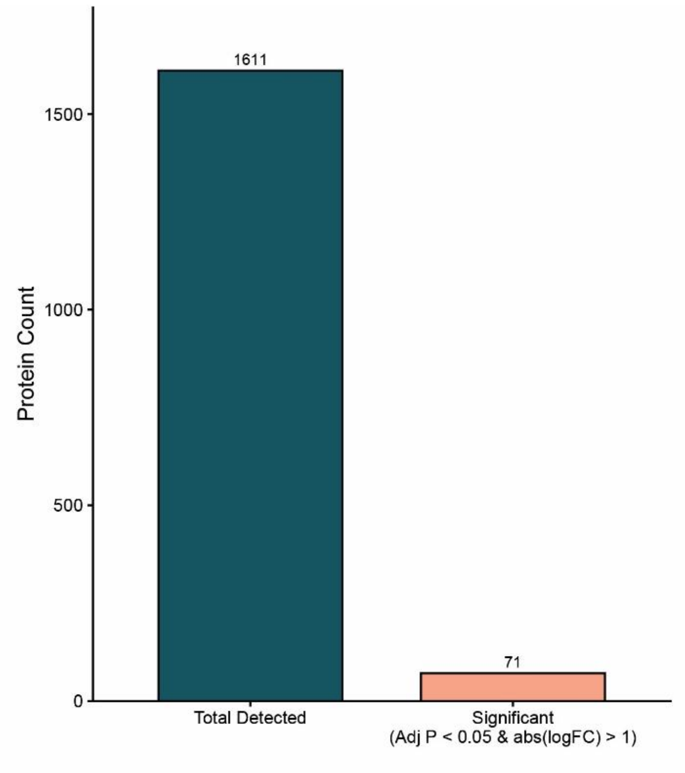

Figure S2: Summary of proteomic quantification results, including total proteins detected across samples and statistically significant different proteins (adjusted $P < 0.05$ and $\log_2$ fold change>1).

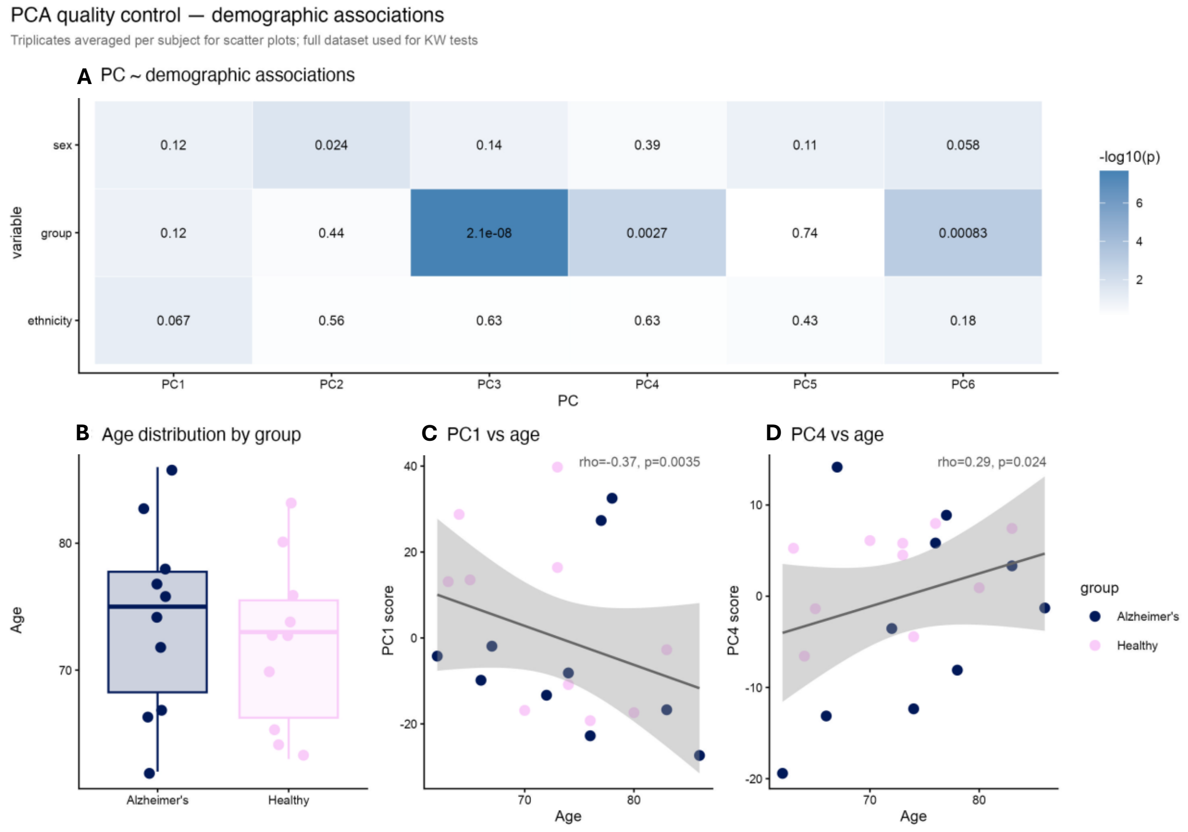

Figure S3: PCA demographic association analysis of MXene–PC proteomics data. (A) Heatmap showing statistical associations between principal components (PC1–PC6) and demographic variables including disease group, sex, and ethnicity. PC3 and PC4 showed the strongest association with disease group. (B) PCA quality control and demographic distribution analyses including age distribution and correlation of principal component scores with age.
